# A journey through the history of PEGylated drug delivery nanocarriers

**DOI:** 10.1007/s13346-024-01608-8

**Published:** 2024-05-25

**Authors:** Ana M. López-Estevez, Ruxandra Gref, Maria J. Alonso

**Affiliations:** 1 CIMUS Research Institute and IDIS Research Institute, Santiago de Compostela, Spain; 2 Department of Pharmacy and Pharmaceutical Technology, University of Santiago de Compostela, Santiago de Compostela, Spain; 3 Institut of Molecular Sciences, Université Paris-Saclay, CNRS, ISMO UMR 8216, Orsay, France

**Keywords:** Nanoparticles, PEGylation, Drug targeting

## Abstract

This note aims to inspire through providing a personal view of the development and potential Drug Delivery Nanocarriers functionalized with polythyleneglycol (PEG). This polymer has been used extensively in Pharmaceutical Technology in a variety of compositions, including polyethylene oxide (PEO)-based surfactants. However, the concept of PEGylation, which started in the 70’s, differs from the functionality of a surfactant, already discloses in the 50’s. Here, we strictly adhere to the biological functionality of PEGylated nanocarriers intended to have a reduced interaction with proteins and, therefore, modify their biodistribution as well as facilitate their diffusion across mucus and other biological barriers. We analyze how this concept has evolved over the years and the benefit obtained so far in terms of marketed nanomedicines and provide the readers with a prospect view of the topic.

## Introduction

The development of surface-engineered drug delivery carriers has been a significant focus in pharmaceutical research, aiming to improve drug efficacy, minimize side effects, and enhance patient compliance. Among the various strategies employed, PEGylation has emerged as a cornerstone technique in the field. This note delves into the fascinating history of PEGylation, tracing its origins, milestones, and impact on drug delivery carriers. In the context of this note the concept of PEGylation refers to the functionalization of drug nanocarriers with the objective of modifying their functionality. Although a number of polyethylene oxide surfactants have been classically used in pharmaceutical technology we will avoid their description while focusing on the PEGylation of lipids, polymers and proteins as a way to change the functionality of nanocarriers and, mainly, improve their capacity to overcome biological barriers and the ir biodistribution.

The roots of PEGylation can be traced back to the 1970s when researchers first explored the conjugation of PEG to proteins. Davis’s team is among the early pioneers who investigated the potential of PEGylation to modify proteins, improving their pharmacokinetics and enhancing their therapeutic properties [1]. In the 1990s, the FDA approval of Adagen^®^ (pegademase bovine), a PEGylated protein therapeutic for the treatment of severe combined immunodeficiency disease (SCID), showcased the clinical potential of PEGylation. This milestone highlighted the ability of PEGylation to improve the pharmacokinetics and efficacy of protein-based drugs, paving the way for future developments in the field.

The application of PEGylation in drug delivery carriers emerged as a promising strategy to overcome challenges associated with rapid clearance and poor biodistribution. By conjugating PEG to liposomes, nanoparticles, and other carriers, researchers could enhance their stability, prolong circulation time, and improve targeting capabilities. The early work on PEGylated liposomes that paved the way for Doxil’s clinical trials in the 1980s primarily involved researchers associated with Sequus Pharmaceuticals, the company responsible for the development of Doxil. One of the key figures involved in this early research was Dr. Alberto Gabizon, who conducted pivotal preclinical studies demonstrating a correlation of liposome circulation time with enhanced cancer drug delivery and efficacy [2], and the first-in-man clinical study demonstrating the pharmacologic advantage of PEGylated liposomal doxorubicin over free doxorubicin [3]. Additionally, Dr. Yechezkel Barenholz expertise in liposome biophysics and drug delivery played a crucial role in the formulation of PEGylated liposomes by optimizing the loading method of doxorubicin [4].

**Fig. 1:**
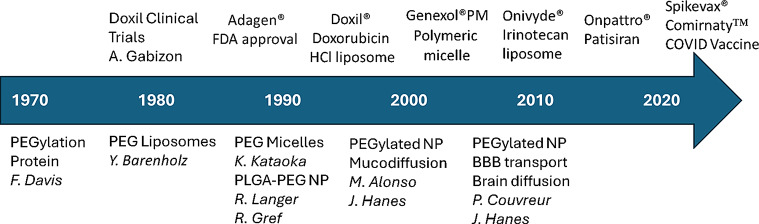
Illustration of some significant events and milestones in PEGylated nanocarriers

In recent decades, PEGylation has continued to evolve with advancements, among which we want to highlight the contribution of PEGylation to modify the biodistribution of nanocarriers, and diffusion across mucus and tissues. Figure 1 illustrates some significant events.

Looking ahead, the field of PEGylation holds promise for addressing unmet medical needs and advancing therapeutic interventions. With ongoing research and technological advancements, PEGylation is poised to remain a cornerstone technique in pharmaceutical development, shaping the future of medicine.

## PEGylation to modify biodistribution and fate of nanocarriers

Through spatial and temporal controlled drug delivery, injectable nanocarriers have the potential to revolutionize disease treatment. Specifically, localizing the release of potent but toxic drugs only at therapeutic sites can lower the overall systemic dose and mitigate damage related to free drugs. Temporally controlling drug release also helps in decreasing unwanted side effects. However, to realize these desired benefits, nanocarriers must remain in the bloodstream long enough to reach or recognize their therapeutic site of action. Effective biodistribution and drug delivery pose challenges as nanocarriers encounter both physical and biological barriers, including stability issues due to shear forces, protein adsorption, and eventual rapid clearance.

In the early 1980s, it was observed that once administered in the bloodstream, nanoparticles tend to be rapidly covered with opsonins, such as complement proteins and immunoglobulins, triggering their recognition and subsequent removal from circulation by the reticuloendothelial system [5]. Consequently, nanocarriers accumulate primarily in the liver and spleen within minutes after administration. Since then, scientists worldwide have made efforts to engineer the surface of nanocarriers with “stealth” coatings, PEG-based being the most widely employed.

Pioneering studies in the 1990s with PEG- and adriamycin-conjugated micelles in Kataoka’s team [6] showed an efficient suppression of tumor growth and increased survival. Also in the 1990s, seminal studies in Langer’s laboratory resulted in the development of biodegradable PLGA nanoparticles coated with PEG brushes resulting from di-or multiblock amphiphilic copolymers PEG-PLGA and PEG_3_-PLGA [7, 8]. PEGylated nanocarriers circulate for hours in the bloodstream rather than minutes [9]. Key factors influencing the fate of PEGylated nanocarriers include PEG surface density and molar mass [10]. For instance, a dense PEG brush with distances between terminally attached PEG chains of less than 2 nm proved most effective in avoiding protein adsorption and recognition by macrophages [11]. Additionally, PEG chains with molar masses higher than 2 KDa were necessary to ensure effective protection. These findings were consistent with theoretical calculation in the team of Pr. Gilles de Gennes [12]. Various methods were deployed to characterize PEG coatings on different types of nanocarriers, including visual demonstrations of the protein-rejecting capabilities of PEGylated nanoparticles [13].

Considerable effort has been dedicated to coupling targeting ligands onto the surface of “stealth” nanocarriers. Typically, ligands such as antibodies, peptides, integrin ligands, glucose, transferrin, and folic acid are coupled at the end of the PEG chain [9]. However, there exists a delicate balance between residence time in circulation and cellular uptake. The “stealth” PEG corona may indeed hinder interactions with targeted cells. One example to circumvent this drawback are nanocarriers with labile PEG coronas: in the tumor microenvironment, the PEG coating detaches, exposing a cell-penetrating peptide [14]. Such systems can adapt properties to optimize delivery based on the current barrier they encounter.

## PEGylation to improve difussion across mucosal barriers

**Fig. 2:**
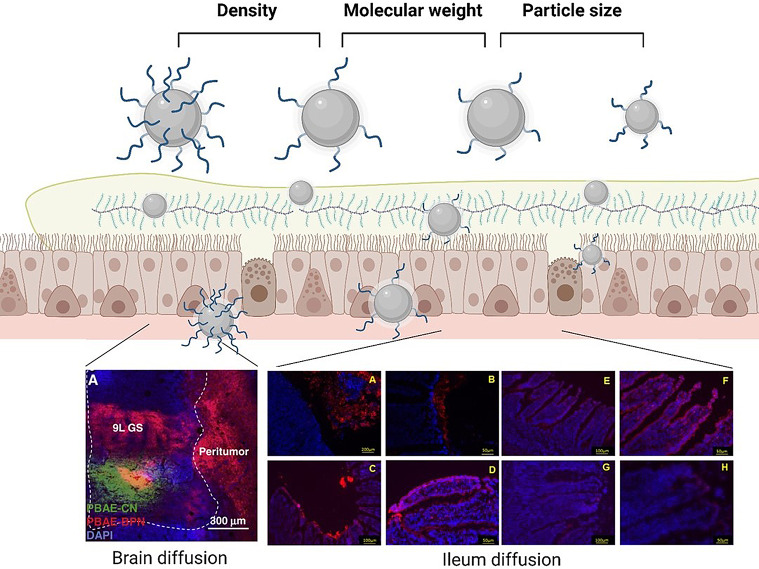
Illustration of the parameters that determine the muco-penetrating properties of nanoparticles, highlighting the PEG coating density, PEG molecular weight and particle size. Brain diffusion image of the distribution of non-PEGylated NPs (PBAE-CN) and PEGylated-NPs (PBAE-BPN) in the glioma-bearing rat. DAPI (blue) represents cell nuclei and yellow indicates co-localization of NPs [15]. Ileum diffusion image of PEGylated NPs with increasing PEG molecular weights [naked (**A**-**B**), 2 kDa (**C**-**D**), 6 kDa (**E**-**F**) and 10 kDa (**G**-**H**)] and their distributions within the mucosa by fluorescence microscopy. Ileum mucosa distributions except for A, stomach mucosa [16]. Figures have been created with BioRender.com

Our laboratory was the first to demonstrate that PEGylation of nanocarriers may enhance their diffusion across mucosal surfaces. It was in the late 1980s when we established that PEGylation played a crucial role in the transport of a protein antigen (tetanus toxoid) encapsulated in PLA-PEG nanocarriers across the nasal mucosa [17]. Subsequently, we identified that both, the size and PEG coating density, were pivotal factors influencing the transport of PLA-PEG nanoparticles across the nasal mucosa [18]. This was substantially corroborated by several authors (Fig. 2).

Building upon this early work, several groups investigated the significance of PEGylation in enabling nanoparticles to overcome other mucosal barriers, such as the intestinal barrier. In particular, we observed that, following oral administration, PLA-PEG exhibited an enhanced stability in digestive fluids and an improved access of the antigen to the blood and lymphatic circulation [19]. Similarly, other authors, among them Irache and colleagues, revealed that coating poly(anhydride) NPs with PEG produced nanocarriers with mucus-permeating properties, influenced by both, the molecular weight and surface density of PEG. However, it was noted that above a certain PEG density, the mucus-permeating properties were reduced, possibly due to a less flexible coating which could get entangled with the macrogol chains [16].

In the early 2000s, our laboratory also explored the impact of PEGylation on the interaction of nanocarriers with the ocular mucosa [20]. Confocal laser scanning microscopy images suggested that PEG coating facilitated the transport of nanocarriers across the entire epithelium, while chitosan coating favored their retention in the superficial layers of the epithelium. Furthermore, investigations by Hanes and colleagues into the movements of polystyrene NPs of various sizes and surface chemistries in fresh bovine vitreous revealed that PEG-coated NPs exhibited uniform rapid diffusion, with similar findings corroborated using cationic PEI-based DNA NPs [21].

Seminal studies conducted in Hanes’ lab further underscored the importance of PEGylation in facilitating diffusion of large polymeric nanoparticles in human cervical (CV) mucus and shielding nanoparticles from mucin interactions [22, 23]. Similar behavior was observed for poly(sebacic acid)-PEG NPs and polystyrene-PEG NPs, suggesting their potential for drug delivery to the lungs, particularly in the context of cystic fibrosis [24, 25].

## PEGylation to improve access and difussion across the brain

There is extensive work in the field of PEGylated nanocarriers for drug delivery to the brain. Here, we will simply highlight seminal work that, from our perspective, has opened the scope of their functionality. For example, Couvreur and his colleagues pioneered work on the performance of PEGylated polycyanoacrylate nanoparticles in crossing the blood-brain barrier (BBB). They hypothesized that passive diffusion across the compromised barrier and macrophage uptake in inflammatory lesions underlie the mechanisms of such particles’ penetration [26]. In another study, the same authors found that the prolonged circulation of nanoparticles in plasma facilitated their preferential accumulation within the tumor, attributed to diffusion/convection-mediated extravasation across the compromised BBB [27]. In another example, chitosan nanospheres conjugated with PEG bearing the OX26 monoclonal antibody, with an affinity for the transferrin receptor, were reported to diffuse into the brain interstitium [28].

Hanes’ Laboratory has also lead the examination of the effect of PEGylation on the diffusion of nanoparticles across the brain. Densely PEG-coated PS NPs, as large as 114 nm, could rapidly penetrate ex vivo human and rat brain tissue. The formation of PEG coatings in the “brush” mode was reported to facilitate diffusion across the brain [29]. PLGA-PEG NPs (69 nm) rapidly diffused in brain tumor tissue, while uncoated PLGA NPs (88 nm) were adhesively immobilized. The enhanced diffusion was translated into an improved efficacy in a rat model of malignant glioma [30]. In a different work it was also shown that DNA-loaded PBAE-PEG (5 K) NPs diffused rapidly in fresh tissues and achieved widespread transgene expression in vivo, in an orthotopic rat brain tumor model [15]. Similarly, PEG(5 K)-poly(aspartic acid) NPs diffused significantly faster than uncoated NPs in both healthy and tumor-bearing brain tissues. These ex vivo studies were consistent with the in vivo distribution, where the volume of distribution of PEG(5 K)-NPs was 29-fold higher than that of uncoated NPs when administered by convection-enhanced delivery (intracranial administration) [31].

In summary, the surface modification of nanocarriers with PEG chains has consistently shown to enhance diffusion across biological surfaces, including mucus-associated barriers and tissues, particularly the brain. It is essential to carefully modulate the molecular weight and surface density of PEG, considering factors such as particle size and the specific biological barrier being targeted.

## PEGylated nanomedicines in the market and prospect view

As elaborated in this Inspirational Note, the application of PEGylation has garnered significant attention due to its capacity to extend circulation time and facilitate the transport of nanocarriers across biological barriers. While its impact on drug/enzyme conjugation is well-documented within the pharmaceutical industry, the utilization of PEGylated nanocarriers remains somewhat limited, albeit with notable exceptions (refer to Table 1 for a non-exclusive list) [32, 33]. Particularly noteworthy is the emergence of PEGylated lipid nanoparticles (LNPs) for RNA delivery, notably in mRNA vaccines, representing a significant milestone. The clinical success of these vaccines not only underscores the potential of PEGylated nanocarriers but also paves the way for their broader clinical application, especially in mRNA therapeutics. Furthermore, the successful large-scale production, distribution, and administration of billions of doses of PEGylated NPs underscore their potential utility on a global scale.

The behavior of intramuscularly injected mRNA vaccines provides valuable insights into the role of PEG in enhancing diffusion. However, a critical area necessitating further investigation concerns the interaction of nanocarriers with proteins and other biological components within the human body. While it has been presumed that PEGylation mitigates such interactions, empirical evidence suggests that factors such as PEG detachment and the affinity of PEGylated nanocarriers for specific proteins require nuanced consideration. Consequently, additional fundamental research is warranted to elucidate optimal strategies for leveraging PEGylated biomaterials to their fullest potential. It is our contention that the forthcoming years will witness the emergence of novel PEGylated nanocarriers, fostering advancements in therapeutic modalities, particularly those centered on biological drugs.

**Table 1:** Approved PEGylated nanomedicines

Trade name [generic name]	Company	Drug	Delivery system	Type of PEG (MW, kDa)	Indication	Adm. route	Agency (year of approval)
Spikevax^®^ [COVID-19 Vaccine, mRNA]	Moderna	mRNA	LNPs	PEG-DMG (2)	COVID-19	IM	FDA (2022)
Comirnaty™ [COVID-19 Vaccine, mRNA]	Pfizer-BioNTech	mRNA	LNPs	ALC-0159 (2)	COVID-19	IM	FDA (2021)
Onpattro^®^ [Patisiran]	Alnylam Pharmaceuticals	siRNA	LNPs	PEG-c-DMG (2)	Polyneuropathy of hereditary transthyretin-mediated amyloidosis	IV	FDA (2018)
Onivyde™ [Irinotecan liposome]	Merrimack Pharmaceuticals	Irinotecan	Liposome	mPEG-DSPE (2)	Metastatic adenocarcinoma of the pancreas post gemcitabine treatment	IV	FDA (2015)
Doxil^®^ [Doxorubicin HCl liposome]	Schering	Doxorubicin	Liposome	mPEG-DSPE (2)	Ovarian cancer, Multiple myeloma, AIDS-related Kaposi’s Sarcoma	IV	FDA (1995)
Genexol^®^PM [Polymeric micelle]	Samyang Corporation	Paclitaxel	Micelles	PEG-poly (D, L-lactide)	Breast cancer, Small cell lung cancer	IV	South Korea (2007)

LNPs lipid nanoparticles; PEG-DMG 1,2-dimyristoyl-rac-glycero-3-methoxypolyethylene glycol-2000; PEG-c-DMG (R)-methoxy-PEG2000-carbamoyl-di-O-myristyl-snglyceride; MW molecular weight; ALC-0159 2-[(polyethylene glycol)-2000]-n, n-ditetradecylacetamide; mPEG-DSPE N-(carbonyl-methoxypolyethylene glycol-2000)-1, 2-distearoly-sn-glycero-3-phosphoethanolamine; IM intramuscular injection; IV intravenous injection

Therefore, PEGylation represents a prominent strategy in pharmaceutical technology, offering considerable potential for enhancing the performance of drug nanocarriers within biological systems. Despite its widespread adoption, the translation of PEGylated nanomedicine candidates into clinical applications has encountered certain challenges. Notably, immunogenicity has emerged as an issue in recent years, as evidenced by reported cases of PEG-related allergic reactions linked to the Pfizer/BioNTech BNT162b2 and Moderna mRNA-1273 vaccines. However, it is crucial to acknowledge that while such reactions warrant regulatory attention, they are not exclusive to PEG and are shared by various excipients. Consequently, while deserving of scrutiny, these events do not justify the exclusion of PEG from formulation technologies. Indeed, a myriad of surfactants and biomaterials containing PEG derivatives are already established within the market. Additional challenges are associated with the chemical conjugation of PEG to various nanocarriers and the potential for their unexpected detachment following in vivo administration Inhibition of acute complement responses towards bolus-injected nanoparticles using targeted short-circulating regulatory proteins [34].
